# Use of Health Apps by Nurses for Professional Purposes: Web-Based Survey Study

**DOI:** 10.2196/15195

**Published:** 2019-11-01

**Authors:** Miguel Angel Mayer, Octavi Rodríguez Blanco, Antonio Torrejon

**Affiliations:** 1 Research Programme on Biomedical Informatics. Hospital del Mar Medical Research Institute Universitat Pompeu Fabra Barcelona Spain; 2 Col·legi Oficial d'Infermeres i Infermers de Barcelona Barcelona Spain

**Keywords:** nurse’s role, smartphones, mobile phone, mobile apps, mHealth

## Abstract

**Background:**

In the last few years, the number of mobile apps for health professionals has increased exponentially. Nevertheless, there is a lack of knowledge about the professional use, training requirements, and quality perception of these apps among health care professionals such as nurses. Considering that the nursing profession is the largest segment of health care workforce in many countries such as Spain, the impact of the use of health apps by these professionals can be critical to the future of modern health care.

**Objective:**

The main objective of this study was to determine if nurses were using health apps professionally and what types of apps they were using. The secondary objectives were (1) to find out if, among nurses, there is a need for training in the use of health apps and (2) to explore nurses’ perceptions of health professional apps, determining whether there is a need for a certification process for health apps and the type of institution or organization that should review and validate these apps for professional use.

**Methods:**

After an initial piloting survey, all registered nurses at the Nursing Association of Barcelona were invited to participate in a 34-item online survey. Eventually, 1293 nurses participated in the survey; however, 52 did not complete the survey properly, omitting both age or gender information, and they were excluded from the analysis.

**Results:**

About half of the respondents (600/1241, 48.35%) had health professional apps installed on their devices and were included for analysis. Most participants in the survey were women (474/600, 79.0%) and the remaining were men (126/600, 21.0%). The most popular types of apps used and installed among nurses were related to drug information, health calculators, and health guidelines. Overall, 97.0% (582/600) of nurses thought that the health apps should be certified, and 80.0% (480/600) agreed that the certification process should be carried out by professional or health institutions. Furthermore, 14.5% (87/600) of participants mentioned that they were asked by their patients to prescribe a health app and only 6.5% (28/430) recommended them often. Most nurses (354/433, 81.8%) who answered the question about the importance of receiving specific training on using and prescribing health apps considered this point a very relevant issue.

**Conclusions:**

About half of the nurses in Catalonia use health apps for professional purposes, and they believe that these types of tools should be validated and certified by health or professional institutions before using them in clinical environments. Although the prescription of health apps in clinical environments is infrequent among nurses, they would be willing to prescribe apps if they were certified by a health organization. Finally, among nurses, there is a need for training in using and prescribing health apps for health care purposes.

## Introduction

### Background

In the last few years, the number of mobile health apps has increased exponentially. The potential of several advantages of using health apps by professionals in clinical environments has been discussed in many forums [1-3]. Currently, there are a lot of apps available to assist health professionals with many different tasks such as health and drug information, communication with patients and other professionals, patient management and monitoring, clinical decision making, and health education and training [4]. In addition, smartphone use by health care professionals is a growing market [5,6].

Several surveys have been carried out to know the use of health apps among general public [7,8], patient associations [9], and some health professionals in hospital settings [10]. In addition, there is a pilot study with the prescription of mobile apps by medical doctors in primary care environments [11]. Moreover, there are also many studies and publications that analyze the quality of health apps, and in most cases, a lack of quality was detected [12-16]. In the recent years, coinciding with the growing number of health apps, diverse organizations and professional institutions have been setting up specific guidelines on the use of health apps addressed to health professionals and students, and above all, among medical doctors [16-20]. This reflects the importance and concerns of the correct use of health apps from legal and ethical points of view.

Nursing practice is developed in many different types of settings such as hospitals, primary care, schools, long-term care facilities, community and public health centers, and homes [21]. Considering that the nursing profession is the largest segment of health care workforce in most countries in the world, including Spain [22,23], the impact of the use of health apps by these professionals is highly likely to be critical to the future of modern health care [24]. Therefore, there is a need for more knowledge about the real use of health apps among nurses in many different clinical environments, including their training requirements and perceptions on the matter. On the basis of this information, health and professional institutions can establish specific measures to improve the use and management of apps in health care settings.

### Objectives

The main objective of this study was to determine if nurses were using health apps professionally and the types of apps they were using. The secondary objectives were (1) to find out if, among nurses, there is a need for training in the use of health apps and (2) to explore nurses’ perceptions of health professional apps, determining whether there is a need for a certification process for health apps and the type of institution or organization that should review and validate these apps for professional use. To the best of our knowledge no previous studies of these characteristics have been carried out focusing exclusively on nurses in Spain. This information will allow us to have a better knowledge of the current use of these technologies by nurses in clinical environments.

## Methods

### Study Design

The study is a descriptive cross-sectional study based on an internet survey of 1293 nurses in Spain. LimeSurvey: GmbH [25], an open source platform, was used to manage the survey online. In October 2017, a pilot study was carried out by sending the survey to 38 nurses to check its proper functioning and to find out if the respondents understood the questions well. The participants in this pilot survey were selected by a convenience method among colleagues in the authors’ workplace. On the basis of the pilot survey, no additional relevant changes were required in the final version of the survey, apart from some typing errors detected in some questions. In March 2018, an email, including a link to the website with the survey, was sent to the 26,907 registered nurses who had a contact email address in the Nursing Association of Barcelona (COIB) of Catalonia [26]. COIB is the second largest nursing association in Spain, with 34,327 registered nurses, after the Nursing Council of Madrid, representing more than 11% of all the registered nurses in this country. The email included information about the purpose of the survey and that the information gathered would be analyzed anonymously. After the first email, 2 more emails were sent as reminders within the following 30 days.

### Survey Items

The list of questions was generated based on the previous experience of the authors in the elaboration of questionnaires addressed to health professionals, such as nurses, physicians, and pharmacists [27,28], and the review of published surveys related to the use of smartphones and health professional apps [7,10].

The survey comprised 34 questions organized in 3 parts: (1) questions related to sociodemographic and professional information such as age, gender, workplace, and professional activity or specialty; (2) information about the use of health apps for professional purposes such as the frequency of use, the type and number of apps installed, and their use; and finally (3) questions and considerations about the quality and certification of health apps and training needs for using health apps for professional purposes. Descriptive statistics were applied for all the items in the survey. Pearson chi-square tests examined the differences in questions with 3 or more options, comparing differences in categorical data among groups, and a Student *t* test was used for comparing continuous variables such as age. Statistical significance was taken at a level of *P*<.05. Questions with open-ended responses or free text, such as the mention of the 3 most frequent apps used or the reasons why they did not prescribe apps, were examined manually, annotating its content. Owing to the exploratory nature of the study, no inferential statistics were applied.

The study was approved by the governing board of the COIB. The analysis of the data was performed using the R programming language for statistical computing and graphics, version 3.4.2 (R Development Core Team).

## Results

### Demographic and Professional Characteristics

The number of respondents to the survey was 1293 (1293/26,907, 4.80%); however, 52 did not provide complete basic information in the survey, such as age or gender, and they were not included in the analysis. With regard to the use of health apps, 51.65% (641/1241) of nurses answered that they did not have health professional apps installed, and the main reasons for not installing these types of apps were (1) lack of knowledge of health professional apps and (2) as they did not use them or they considered that the health apps were not useful for health purposes. Other less frequent reasons were the fact that their professional activity did not involve contact with patients, existence of technical limitations with their mobile phones, or mistrust of health apps. In this group, 85.6% (549/641) were women and 14.4% (92/641) were men. The mean age was 44.96 (SD 13.45) years.

Overall, 48.35% (600/1241) of the respondents had health professional apps installed on their devices. In this group, 79.0% (474/600) were women and 21.0% (126/600) were men, with statistically significant differences with regard to the group of nurses who had not health professional apps installed (*P*<.001). The average age of the respondents who had installed health apps was 43.12 (SD 11.32) years, and there were no significant differences between the ages of men and women (*t*_160.51_=−1.5078; *P*=.13. The distribution of age was 16% baby boomers (>56 years), 58% generation X (35-56 years), and 26% millennials (<35 years). With regard to the professional area, most nurses came from hospital care (288/600, 48.0%) and primary care (144/600, 24.0%). With regard to the specialization, the most frequent groups were general nursing (318/600, 53.0%) and medical-surgical nursing (84/600, 14.0%). The complete distribution of gender, age, nursing specialties, professional activity, and the area of work of the nurses who had installed and were using health professional apps can be seen in Table 1.

**Table 1:** Demographics of surveyed nurses who had health professional apps installed, in terms of gender, age, specialty, and professional area (N=600).

Demographics		Values, n (%)
**Gender**		
	Male	126 (21.0)
	Female	474 (79.0)
**Age (years)**		
	Millennials (<35)	156 (26.0)
	Generation X (35-56)	348 (58.0)
	Baby boomers (>57)	96 (16.0)
**Nursing specialties**		
	General	318 (53.0)
	Midwifery	30 (5.0)
	Mental health	18 (3.0)
	Pediatrics	48 (8.0)
	Family and community	42 (7.0)
	Occupational health care	36 (6.0)
	Medical-surgical	84 (14.0)
	Geriatrics	30 (5.0)
**Professional activity**		
	Hospital care	288 (48.0)
	Primary care	144 (24.0)
	Social health care	42 (7.0)
	Management	30 (5.0)
	Prehospital care	24 (4.0)
	Teaching/research	18 (3.0)
	Private practice	18 (3.0)
	Other	42 (7.0)
**Area of work**		
	Urban	565 (94.2)
	Rural	35 (5.8)

### Health Apps Usage

Overall, nurses prefer smartphones (489/536, 91.2%) instead of tablets (47/536, 8.8%) for managing apps professionally. Most devices were less than 2 years old (413/536, 77.0%), and 42.7% (229/536) of the devices were less than a year old. As for the operating system, 51.1% (274/536) had Android and 45.7% (245/536) had iOS. Furthermore, 57.1% (300/525) of the nurses had organized health professional apps in specific folders in their mobile phone.

**Figure 1:**
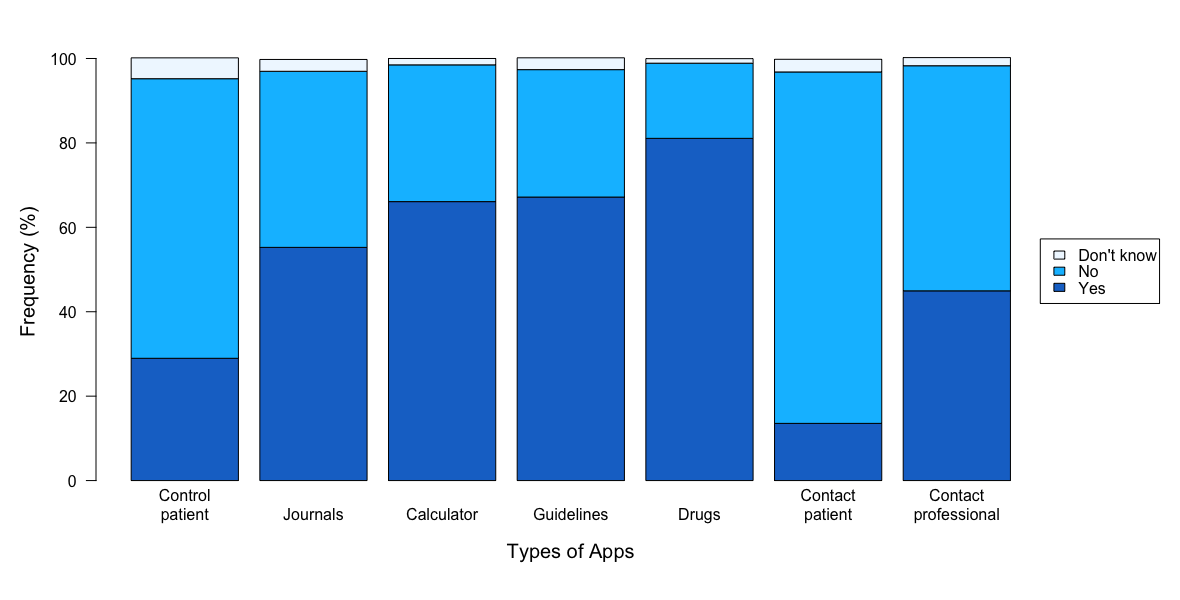
Most frequent types of apps used by nurses.

Most nurses had installed between 2 and 5 apps; 65.4% (344/526) used 1 health professional app at least once a week, 30.6% (161/526) used between 2 and 5 apps, and 3.2% (17/526) used more than 5 health apps. The number of apps installed depended on the gender of the participants; men had more health apps installed than women (χ^2^_2_=12.65; *P*=.002), and at the same time, they used apps more frequently than women (χ^2^_2_=9.30; *P*=.009). Age groups did not significantly differ with regard to the number of apps installed on the devices (χ^2^_4_=7.77; *P*=.10), although baby boomers tended to have more professional apps installed than the other age groups. Age group was not a factor that determined the number of health apps used (χ^2^_2_=5.21; *P*=.27). The most popular types of apps installed and used were related to drug information among 81.1% of nurses (377/465), health guidelines among 67.0% (313/467), health calculators among 66.1% (308/466), and communication with patients among 13.5% (63/465; see Figure 1).

### Prescription of Health Apps

Overall, 97.0% (582/600) of nurses thought that health apps should be certified and 80.0% (480/600) agreed that the certification process should be carried out by professional or health institutions. Most nurses who answered the question related to the prescription of apps never prescribed a health professional app (266/430, 61.9%). If the apps were certified by a health or scientific organization, 50.4% (134/266) of nurses who answered this question would be willing to prescribe them. The willingness to prescribe certified apps was not different between genders (χ^2^_2_=3.66; *P*=.16) and among age groups (χ^2^_4_=3.04; *P*=.55). The recommendation of apps by nurses to colleagues did not show significant differences among age groups (χ^2^_4_=4.28; *P*=.37) or gender (χ^2^_4_=3.96; *P*=.14). Table 2 shows the list of questions and responses related to the features and use of health apps for professional purposes, and Table 3 shows the questions related to different aspects of the prescription of apps (see Multimedia Appendix 1 for the English version of the full list of items and responses).

**Table 2:** Questions and answers, including information about the features and use of health apps by nurses for professional purposes.

Survey question and response options			Answers received, n (%)
**How many apps do you have installed on your smartphone? (n=522)**			
	1		154 (29.5)
	2-5		316 (60.5)
	>5		52 (10.0)
**How many apps do you use regularly (at least once a week)? (n=522)**			
	1		344 (65.9)
	2-5		161 (30.8)
	>5		17 (3.3)
**You are using health apps for:**			
	**As a tool for managing patients (n=466)**		
		Yes	135 (29.0)
		No	308 (66.1)
		I don’t know	23 (4.9)
	**Access to scientific journals (n=465)**		
		Yes	258 (55.5)
		No	194 (41.7)
		I don’t know	13 (2.8)
	**As a calculator for doses and scales (n=466)**		
		Yes	308 (66.1)
		No	151 (32.4)
		I don’t know	7 (1.5)
	**Clinical guidelines and protocols (n=467)**		
		Yes	313 (67.0)
		No	141 (30.2)
		I don’t know	13 (2.8)
	**Information about drugs (n=465)**		
		Yes	377 (81.1)
		No	83 (17.8)
		I don’t know	5 (1.1)
	**Communication with patients (n=465)**		
		Yes	63 (13.5)
		No	388 (83.4)
		I don’t know	14 (3.0)
	**Communication with professionals (n=466)**		
		Yes	209 (44.8)
		No	248 (53.2)
		I don’t know	9 (1.9)
**In relation with the use of health apps, indicate the option that is closest to your opinion (n=466)**			
	**They help me solve professional doubts**		
		Never	14 (3.0)
		Sometimes	226 (48.5)
		Often	226 (48.5)
	**In general, they are easy to use**		
		Never	11 (2.4)
		Sometimes	139 (29.8)
		Often	316 (67.8)
	**They facilitate my professional tasks**		
		Never	16 (3.4)
		Sometimes	236 (50.6)
		Often	214 (45.9)
	**They are a support tool for managing my patients**		
		Never	161 (34.5)
		Sometimes	188 (40.3)
		Often	117 (25.1)
**Do you recommend any apps to your colleagues for professional use? (n=430)**			
	Never		62 (14.4)
	Sometimes		283 (65.8)
	Often		85 (19.8)

**Table 3:** Questions and answers about the prescription of health professional apps.

Survey question and response options		Answers received, n (%)
**Currently, do you prescribe apps to your patients? (n=430)**		
	Never	266 (61.9)
	Sometimes	136 (31.6)
	Often	28 (6.5)
**If that’s the case, what is the aim of prescribing apps to your patients? (n=336)**		
	Health promotion	129 (38.4)
	Health information, educational support	98 (29.2)
	Patient self-management and control	64 (19.0)
	Monitoring patients	38 (11.3)
	Others	7 (2.1)
**Would you prescribe apps to your patients if they were certified by a health organization? (n=266)**		
	Yes	134 (50.4)
	No	50 (18.8)
	I don’t know	82 (30.8)
**Did your patients ask for an app to be prescribed to them? (n=430)**		
	Never	297 (69.1)
	Sometimes	128 (29.8)
	Often	5 (1.2)
**Do you think that some health organization, scientific or professional association should certify health apps (both for patients and professionals)? (n=430)**		
	Yes	392 (91.2)
	No	11 (2.5)
	I don’t know	27 (6.3)
**Do you think being trained to use and prescribe health apps could be of interest to you? (n=430)**		
	Yes	354 (82.3)
	No	27 (6.3)
	I don’t know	49 (11.4)
**Do you think the health or professional organization should promote recommendations for developing health apps (addressed to developers)? (n=430)**		
	Yes	377 (87.7)
	No	4 (0.9)
	I don’t know	49 (11.4)

The reasons why surveyed nurses had not recommended health apps to patients were because of the *lack of knowledge* about the type and use of these apps, although there were other factors such as the type of patients, patients with limited capacity to use apps (elderly people), or related to the type of professional activity such as in intensive care units, working in a night shift or geriatrics department, no patient contact, and lack of time. Very few participants (16/266, 6.0%) who answered the question about the reasons why they did not prescribe health apps considered them unnecessary. Furthermore, 59.8% (359/600) answered the question about the 3 most frequent apps they used, and the first 3 most frequent apps mentioned were Infermera virtual (an app developed and promoted by the COIB), Vademecum International (an app containing a handbook for drugs consultation), and 061 CatSalut Respon (an app promoted by the Catalan government for contacting health care services and obtaining health advice). In addition, the most frequent apps addressed to specific diseases were Trata la upp (an app for the management of pressure ulcers), Pukono (an app that includes information and diet recommendations for patients with hypertension), Diabetes a la carta (an app for managing diabetes), SocialDiabetes (an app for managing diabetes), and S’acabó (an app for smoking cessation). Table 4 includes the 16 most frequent apps used by nurses among the 238 apps they mentioned in the survey.

**Table 4:** The 16 most frequently used apps by nurses in Catalonia.

Name of the app	Description	Mentions, n
Infermera virtual	This app is promoted by the Nursing Association of Barcelona and provides a collection of resources, services, and useful information for nurses	92
Vademecum Internacional	This app contains a handbook for drugs consultation and is promoted by Vidal Vademecum Spain	84
061 CatSalut	An app promoted by the Catalan government for contacting health care services and obtaining health advice and information for the general public and health professionals	46
Enfermeria blog	App of a blog with useful information for nurses	24
HealthScience	This app includes the most recent abstracts of scientific publications for health professionals	22
NandaNocNic	This app includes the codes of the North American Nursing Diagnosis Association diagnosis, results, and interventions	21
Medicamentos via parenteral	An app that includes information for the management and administration of parenteral drugs	19
iVacunes Gencat	An app promoted by TICSalut of the Catalan government that includes information about vaccines and other related resources	17
aempsCIMA	This app offers information about all the authorized drugs in Spain and is promoted by the Spanish Medicines Agency	15
DosisPedia	An app for the management, administration, and doses of drugs	13
Trata la upp	An app for the management of pressure ulcers	12
ECG Práctico	An app of the Spanish Cardiology Society with guidelines for the interpretation of the electrocardiogram,	11
Babymecum	An app for the management of drugs doses and administration in pediatrics	10
GuiaSalud	This app includes the most important clinical guidelines and protocols of the Spanish National Health Service	10
iDoctus	This app includes information on drugs and diseases, several dosage calculators, and drug-drug interactions	8
eMerMed Science	An app that includes the most recent abstracts of scientific publications for emergency medicine professionals	7

Only 14.5% (87/600) of the participants mentioned that they were asked by their patients to prescribe a health app to them. The most frequent apps prescribed were related to diet and exercise (19/87, 21.8%), diabetes (13/87, 14.9%), health tips (10/87, 11.5%), hypertension (8/87, 9.2%), and smoking cessation (8/87, 9.2%). With regard to the importance of receiving specific training in using and prescribing health apps, 72.2% (433/600) of nurses answered this question. Most of them (354/433, 81.8%) answered that it was necessary to receive specific training in the matter, without significant differences among age groups (χ^2^_4_=3.40; *P*=.35) or gender.

## Discussion

### Principal Findings

First, just under half of the nurses who participated in the survey used health professional apps. The main reasons for not using health apps were the lack of knowledge or interest. In only a few cases did they consider these apps unnecessary for carrying out their jobs. It is worth noting that the most frequent apps used by nurses were promoted by health institutions of reference such as the Health Department of Catalonia and the COIB and that these apps are used to support their professional activity and patient advice. It is surprising that older nurses (baby boomers) tend to have more apps installed than the younger nurses, considering that in general, young people are more used to incorporating new information and communication technologies than older people. Baby boomers, who have more professional experience, usually did not require the help of apps for drug information and for calculating doses in clinical care. In addition, on the basis of their professional experience, they were willing to use health professional apps for patient control or to be in touch with them. On the contrary, younger nurses consult health professional apps more frequently to calculate drug posology and less frequently to establish communication with patients. Perhaps this is because of a lack of confidence in using apps for monitoring patients. Considering the number of apps installed by gender, okmen had more apps installed on their devices than women. Baby boomers tended to have more professional apps installed than the other age groups.

Second, the prescription of health apps is not common among health professionals, and one reason for that could be the fact that this activity is not yet totally regulated. Nevertheless, nurses would be willing to prescribe apps more frequently if they were certified by a health or scientific organization. This is the first study that explores the opinion of health professionals, particularly nurses, about the need of certifying health apps. The prescription of apps in clinical settings requires several considerations; on the one hand the importance of using health apps of high quality and those that have been previously tested are among the most relevant ones. On the other hand, it is necessary to guarantee their technical and organizational integration in a health care system, based on specific protocols [11]. If health apps are to be adopted, patients and health professionals must be sure that they are safe and effective [29]. With regard to the types of apps prescribed, this aspect may be explained considering that the conditions for which these apps are prescribed are among the most common chronic diseases, and at the same time, there are a lot of apps in the market focusing on smoking, exercise, and diabetes control.

Finally, our findings also suggest that currently the prescription and use of health apps is not frequent among certain patients that health professionals in general are taking care of, particularly elderly people. In this sense, the current aging population requires strategies to promote the use of health apps among this population. One of the possible solutions is to facilitate the training of the elderly in the use of mobile technologies. This population and health professionals can benefit from the use of health apps for monitoring and managing these patients suffering from chronic diseases [30]. In addition, the survey provides us with some insights related to relevant aspects such as the need to train nurses in the use and prescription of health professional apps in clinical environments and also the importance of having some guidelines to be able to select apps of high quality [31-33]. This implies that evaluation tools and protocols should be applied before deciding on the introduction of apps in clinical settings. Professional and health institutions should have a key role in these types of activities, promoting training programs and the evaluation of the quality and usefulness of specific health apps in clinical environments.

In the last few years, the evolution of mobile phones and other devices, as well as the apps developed, has happened at an unprecedented rate. Nurses, and health professionals in general, must be trained to use information and communication technologies such as mobile apps, smart wearable technologies, or telemedicine apps [4,24,34,35]. For this reason, it is critical to have information about how these professionals use and view the utility of these apps. This study examined the health app usage among registered nurses from the second most important nursing association of Spain.

### Strengths and Limitations of the Study

On the one hand this study has several strengths. First, this is the first time that this type of survey has been performed among nurses in Spain, the third largest country in Western Europe, with a health system that is almost universal (covering 99.1% of the population) and the highest life expectancy in the European Union [36]. Second, although the rate of participation is low in absolute terms, the number of participants is quite high, and included all the different nursing specialties. Finally, original aspects related to the use and certification of health professional apps were analyzed. On the other hand, this study presents some limitations. The number of participants in the survey was very low compared with the number of nurses who received the survey. In addition, the number of men was significantly higher than the group of nurses who did not respond to the survey. For these reasons, the results could overestimate or underestimate the real use and the interest of nurses in health apps and the influence of some factors such as gender, introducing a potential selection bias, which is very common in this type of online survey. We should take into account that the survey was sent to nurses who had an email address; however, 21.73% (7471/34,378) of the registered nurses did not have a contact email address or did not use a COIB email address, and they did not receive the questionnaire.

### Conclusions

The use of health professional apps was not generalized among nurses at the time of the survey and it was very unusual for patients to ask for a health app. The most popular apps are used to support the professional activity of nurses, and most of them are promoted by professional and health institutions. In addition, the prescription of health apps is not common among nurses, but almost all nurses believed that health apps should be certified by health institutions or professional associations. According to the results of the survey, the certification of health apps would be a very important deciding factor in the prescription of apps to patients in clinical environments. Moreover, nurses expressed their interest in and their need for specific training in the use and prescription of health apps, indicating the potential impact of the introduction of these technologies in clinical environments. On the basis of the results of the survey, COIB will consider the possibility of giving nurses some training in the use and prescription of health apps in clinical environments. On the basis of the concerns about the quality of apps that nurses expressed, COIB will consider setting up some specific guidelines to rate the quality of health apps.

## Abbreviations

COIB: Nursing Association of Barcelona

## Appendix

Multimedia Appendix 1 Health professional apps survey.
