# Correction: Use of Health Apps by Nurses for Professional Purposes: Web-Based Survey Study

**DOI:** 10.2196/18516

**Published:** 2020-04-30

**Authors:** Miguel Angel Mayer, Octavi Rodríguez Blanco, Antonio Torrejon

**Affiliations:** 1 Research Programme on Biomedical Informatics. Hospital del Mar Medical Research Institute Universitat Pompeu Fabra Barcelona Spain; 2 Col·legi Oficial d'Infermeres i Infermers de Barcelona Barcelona Spain

In the manuscript “Use of Health Apps by Nurses for Professional Purposes: Web-Based Survey Study” (JMIR Mhealth Uhealth 2019;7(11):e15195) there was an error in the original published PDF. The original published PDF version of the manuscript available on the JMIR mHealth and uHealth website displayed the title as “Use of Health Apps by Nurses for Professional Purposes in Catalonia, Spain: Web-Based Survey Study”. The correct title is “Use of Health Apps by Nurses for Professional Purposes: Web-Based Survey Study”. The PDF has now been revised to remove “in Catalonia, Spain”. This correction notice will appear on the JMIR mHealth and uHealth website on April 30, 2020. Because the XML had been correctly submitted to all other repositories, it will not need to be resubmitted.

