# Supplementary material for: Use of Health Apps by Nurses for Professional Purposes: Web-Based Survey Study
Source: JMIR Mhealth Uhealth. 2019 Nov 1;7(11):e15195. doi: 10.2196/15195 (PMC6858615; doi:10.2196/15195)
Supplement: Multimedia Appendix 1 [file mhealth_v7i11e15195_app1.pdf]

# Health Professional Apps Survey

Survey about health apps and their use among nurses for professional purposes.

Among the objectives of the Information System Technologies of the Nursing Association of Barcelona (COIB) are, knowing the use of health professional apps by nurses and covering the training needs in relation with the use of these apps. With these objectives, we are asking you to answer the following questionnaire that will only take some few minutes to fill in.

## Additional information

- **Health apps definition:**
  Health apps are application programs that offer health-related services for smartphones and tablets.

- **Prescription of apps:**
  Health apps can be recommended in clinical environments and used as a way to monitor or to give guidelines to patients

**Contact support:** Laura Rausell (lrausell@coib.cat)

The researchers thank you the participation in the survey.

**Researchers:**
Miguel A. Mayer
Octavi Rodríguez-Blanco
Toni Torrejón

This survey is anonymous and does not gather any kind of personal information such as your name, surname or professional id, and it contains 34 questions.

## Introductory question:

- **Do you have health professional apps installed in your mobile phone or tablet?**
  Select one:
  - Yes
  - No

If your answer of the previous question was “No”, please answer the following question:

- **Could you tell us the reasons why you do not have health apps installed in your mobile phone or tablet?**
  Write down the answer here (free text):

## Personal and professional data

- **Month and year of birth:**

- **Gender:**
  - Female
  - Male

- **Specialty:**
  - General
  - Midwifery
  - Mental health
  - Pediatrics
  - Family and Community
  - Occupational healthcare
  - Medical-Surgical
  - Geriatrics

- **Professional activity:**
  - Hospital care
  - Primary care
  - Social healthcare
  - Management
  - Pre-hospital care
  - Teaching/Research
  - Private practice
  - Other

- **Area of work:**
  - Urban
  - Rural

## Use of Information and Communication Technologies

- **What kind of device do you use for professional purposes more often?**
    - Smartphone
    - Tablet

- **What kind of social media platforms for professional purposes do you have?**

  Tick the answer that is closer to your use for each social media platform:
  I don't use it /Once a month /Once a week /Once a day /Several times a day
    - Facebook
    - Twitter
    - Linkedin
    - Instagram
    - Whatsapp
    - Skype
    - Google+/Hangouts
    - Youtube or Vimeo

- **How much time does your device have?**
    - Less than 6 months
    - Between 6 months and 1 year
    - Between 1 and 2 years
    - Between 2 and 3 years
    - More than 3 years

- **What is the OS of your device?**
    - iOS
    - Android
    - Windows Phone
    - Other

- **What kind of Internet connection do you usually use in your device?**
    - 3G or 4G
    - I need to connect to a Wifi
    - I use either 3G, 4G or Wifi

- **Do you use some of the following collaborative platforms to share information with your colleagues?**
    - o Amazon Cloud Drive
    - o Apple iCloud
    - o Box

- Dropbox
    - Google Drive
    - Microsoft OneDrive

- **Do you use some of the following collaborative platforms to share information with your patients?**
    - Amazon Cloud Drive
    - Apple iCloud
    - Box
    - Dropbox
    - Google Drive
    - Microsoft OneDrive

## Information about the use of health professional apps

- **Do you have health professional apps organized in folders in your device?**
    - Yes
    - No

- **In relation with apps for communication, indicate which of the following you are using them for being in touch with your patients?**
    - Facebook
    - Twitter
    - LinkedIn
    - Instagram
    - WhatsApp
    - Telegram
    - Skype
    - Google+/Hangouts
    - Youtube or Vimeo

- **In relation with apps for communication, indicate which of the following you are using them for being in touch with your colleagues?**
    - Facebook
    - Twitter
    - LinkedIn
    - Instagram
    - WhatsApp
    - Telegram
    - Skype
    - Google+/Hangouts
    - Youtube or Vimeo

- **Do you use some apps with push notifications for receiving professional information of your interest?**
    - Yes
    - No
    - I don't know

- **Mention the three health professional apps that you are using the most.**
    - App1 (free text)
    - App2 (free text)
    - App3 (free text)

- **If you have decided to prescribe a health professional app it was because:**
    - A colleague recommended it to me
    - I used it previously in order to validate it
    - A patient recommended it to me
    - Pharma recommended it to me
    - Apps that I've seen recommended on the Internet
    - Apps included in a specific platform for prescribing them

- **Why don't you prescribe health professional apps to your patients?**
  Write down the answer here (free text):

- **Give us an example of an app you have prescribed:**
  Write down the answer here (free text):

- **Indicate in which health problem of situation patients asked you for prescribing a health app:**
  Write down the answer here (free text):

- **Do you think a health or professional organization should give specific training for designing health apps?**
    - Yes
    - No
    - I don't know

- **How many apps do you have installed on your smartphone?**
    - One
    - Between 2 and 5
    - More than 5

- **How many apps do you use regularly (at least once a week)?**
    - One
    - Between 2 and 5
    - More than 5

- **You are using health apps for :**
    - A tool for managing patients Yes / No / I don’t know
    - Access to scientific journals. Yes / No / I don’t know
    - As a calculator for doses, scales Yes / No / I don’t know
    - Clinical guidelines and protocols Yes / No / I don’t know
    - Information about drugs Yes / No / I don’t know

- **In relation with the use of health apps, indicate the option that is closest to your opinion**:
    - They help me solve professional doubts: Never /Sometimes /Often
    - In general, they are easy to use: Never /Sometimes /Often
    - They facilitate my professional tasks: Never /Sometimes /Often
    - They are a support tool for managing my patients: Never /Sometimes /Often

- **Do you recommend any apps to your colleagues for professional use?**
    - Never
    - Sometimes
    - Often

- **Currently, do you prescribe apps to your patients?**
    - Never
    - Sometimes
    - Often

- **If that’s the case, what is the aim of prescribing apps to your patients?**
    - Health promotion
    - Health information, educational support
    - Patient self-management control
    - Monitoring patients
    - Other

- **Would you prescribe apps to your patients if they were certified by a health organization?**
    - Yes
    - No
    - I don’t know

- **Did your patients ask for an app to be prescribed to them?**
    - Never
    - Sometimes
    - Often

- **Do you think that some health organization, scientific or professional association should certify health apps (both for patients and professionals)**
    - Never
    - Sometimes
    - Often

- **Do you think being trained to use and prescribe health apps could be of interest to you?**
    - Never
    - Sometimes
    - Often

- **Do you think the health or professional organization should promote recommendations for developing health apps addressed to developers?**
    - Never
    - Sometimes
    - Often
